# A Human Brain Model Mimicking Umbilical Cord Mesenchymal Stem Cells for the Treatment of Hypoxic-Ischemic Brain Injury

**DOI:** 10.3390/ijms241814208

**Published:** 2023-09-18

**Authors:** Xidan Li, Haijing Liu, Chao Han, Jianglin Luo, Xin Guan, Liang Wang, Ying Li, Jiayi Wang, Hua Piao, Wei Zou, Jing Liu

**Affiliations:** 1 Stem Cell Clinical Research Center, The First Affiliated Hospital of Dalian Medical University, Dalian 116011, China; lixidandan@163.com (X.L.); liuhaijing1997@163.com (H.L.); hanchao0427@163.com (C.H.); 13440090927@163.com (J.L.); jessie_guan@163.com (X.G.); wangliang@dmu.edu.cn (L.W.); liying_xx@163.com (Y.L.); jiayiwang94@163.com (J.W.); 2 Liaoning Key Laboratory of Frontier Technology of Stem Cell and Precision Medicine, Dalian Engineering Research Center for Genetic Variation Detection of Infectious Pathogenic Microorganisms, Dalian Innovation Institute of Stem Cell and Precision Medicine, Dalian 116085, China; piaohua88@163.com (H.P.); weizou60@126.com (W.Z.); 3 College of Basic Medical Science, Dalian Medical University, Dalian 116027, China

**Keywords:** OGD, microglia, mesenchymal stem cells, central immune microenvironment, interaction, astrocytes

## Abstract

We used an in vitro model of the human brain immune microenvironment to simulate hypoxic-ischemic brain injury (HIBI) and treatment with human umbilical cord mesenchymal stem cells (hUMSCs) to address the transformation barriers of gene differences between animals and humans in preclinical research. A co-culture system, termed hNAME, consisted of human hippocampal neurons (N), astrocytes (A), microglia (M), and brain microvascular endothelial cells (E). Flow cytometry measured the apoptosis rates of neurons and endothelial cells. hNAME-neurons and endothelial cells experienced more severe damage than monolayer cells, particularly after 48 h and 24 h of reoxygenation (OGD48/R24). Western blotting identified neuroinflammatory response markers, including HIF-1α, C1q, C3, TNF-α, and iNOS. Inflammatory factors originated from the glial chamber rather than the neurons and vascular endothelial chambers. A gradual increase in the release of inflammatory factors was observed as the OGD and reoxygenation times increased, peaking at OGD48/R24. The hNAME value was confirmed in human umbilical cord mesenchymal stem cells (hUMSCs). Treatment with hUMSCs resulted in a notable decrease in the severity of neuronal and endothelial cell damage in hNAME. The hNAME is an ideal in vitro model for simulating the immune microenvironment of the human brain because of the interactions between neurons, vessels, astrocytes, and microglia.

## 1. Introduction

The causes of various neurological disorders and their treatment remain largely unresolved. Direct testing of the human brain is often not feasible owing to ethical considerations; therefore, animal (in vivo) or cellular (in vitro) models are commonly used. The successful application of animal experiment results to clinical trials proves challenging because of variations in the subunits and action sites of the same genes among different species. These differences create obstacles in the clinical translation process. In vitro models are widely used in basic experiments; however, they present challenges owing to species differences and difficulties in replicating the in vivo biological characteristics. In addition, the basic cellular structure is limited by the absence of intercellular communication and complex bodily environments. Creating an in vitro model of a multicell co-culture derived from the human brain is, therefore, imperative.

The brain environment is primarily composed of blood vessels, neurons, astrocytes, microglia, and oligodendrocytes. The brain utilizes blood vessels to transport and exchange substances throughout its structure. The central nervous system utilizes neurons with uniform numbers and blood vessel distribution to transmit information [1]. Establishing synaptic connections among neurons is crucial for transmitting information. A major function of oligodendrocytes is the creation of myelin sheaths around neuronal axons. This sheath safeguards neurons and facilitates the rapid transmission of bioelectrical signals. Neurons and oligodendrocytes form nerve conduction tracts that convey instructions from the brain to the periphery.

It was previously believed that the central nervous system is an independent system protected by the blood-brain barrier, allowing it to be “immune privileged” [2]. This means that peripheral substances do not enter the brain, and transplants in the brain tend to last longer than those in the periphery [2]. Recent research has revolutionized our understanding of the brain’s immune privilege. The brain creates lymphatic vessels and immune cells during development, and innate and adaptive immune cells regulate brain function and repair damage in the central nervous system during disease [3,4,5,6]. The microglia in the brain may be few; however, they are essential as resident immune cells that immediately respond to brain injuries. They can change their form, number, and function to adapt to diseased environments. These cells play a crucial role in initiating the innate immune response and promoting inflammation in the central nervous system [7]. Astrocytes are not considered classical immune cells; however, they also play an immunomodulatory role after brain injury and are often activated after microglia activation, contributing to the central adaptive immune response [8]. The interaction between microglia and astrocytes has a significant impact on the progression and prognosis of central nervous system disease [9]. The secretion of complement 1q (C1q), tumor necrosis factor (TNF-α), and interleukin 1 alpha (IL-1α) by microglia leads to the activation of A1 toxic astrocytes. On the other hand, the production of A2-protected astrocytes is encouraged by IL-10 [10,11]. Astrocyte-derived IL-3 binds specific receptors on the surface of microglia in Alzheimer’s disease (AD). This interaction prompts microglia to migrate toward the AD focal Aβ, where they play a phagocytic role in clearing the area [12]. Other studies have shown that microglia and astrocytes are activated in the vicinity of amyloid plaques. Additionally, complement 3 (C3), originating from astrocytes, interacts with microglia C3a receptors, leading to impaired phagocytic function of microglia and the further advancement of Aβ [13]. Microglia and astrocytes play a crucial role in maintaining the environmental balance in the brain and are essential for building in vitro models.

Hypoxic-ischemic brain injury (HIBI) results from the narrowing or blocking of the arteries of the brain (the carotid and vertebral arteries), leading to brain tissue damage. Conditions such as adult ischemic stroke (IS) and cerebral palsy (CP), collectively known as HIBI, are prevalent in neurological clinical practice and have high incidence rates. HIBI causes significant long-term neurological deficits, including hemiplegia, hemianopsia, aphasia, and cognitive impairment, which can cause disability and even death. Therefore, this condition burdens both the family and society [14]. Additionally, individuals who experience a stroke may also suffer from post-stroke depression, sleep disorders, and various mental and psychological complications, all of which can significantly impact their overall quality of life [15]. A Lancet report revealed that HIBI has become the leading cause of death in China [16]. This study focused on HIBI, a prevalent neurological condition, as a representative example of neurological disorders. We utilized the classical treatment for oxygen-glucose deprivation (OGD) in an in vitro model to simulate the pathological changes associated with HIBI, which involved replacing the sugar-free medium and culturing cells in an oxygen-free environment. This model provides a more accurate simulation of central nervous system damage and addresses the issue of gene heterogeneity resulting from species differences.

In the last two decades, stem cell therapy has become one of the most promising methods for repairing CNS nerve injuries, promoting the brain’s natural regenerative potential to restore lost function after HIBI [17]. Mesenchymal stem cells (MSCs) have functions in cell migration, angiogenesis, immune regulation, neuroprotection, and neural circuit reconstruction. MSCs exert neurotrophic effects through paracrine secretion, polarizing damaged neurons, promoting axonal growth, and repairing myelin sheaths, and their use is expected to become one of the most effective strategies for the treatment of HIBI [18]. Our team demonstrated that human umbilical cord mesenchymal stem cells (hUMSCs) can regulate astrocytes, which, in turn, improves brain microenvironment homeostasis. hUMSCs alleviate neuroinflammation from astrocytosis and promotes neuronal plasticity regeneration by inhibiting polypyrimidine tract binding protein-1 (PTBP-1) in HIBI neonatal rats [19]. hUMSCs have been found to be effective in treating spinal cord injuries by reducing tissue cavitation and increasing the number of Nissl bodies in the damaged area. Additionally, hUMSCs decreased the levels of TNF-α, NF-kB, and PTBP-1, while increasing the neuronal marker TUJ1 and oligodendrocyte marker Olig2 [20]. These previous results indicate that hUMSCs may repair nerve injury by regulating the central immune microenvironment. To investigate the sensitivity of the HIBI in vitro model to injury and treatment, we treated the model with hUMSCs in an OGD injury simulation.

## 2. Results

### 2.1. Model Construction and Identification

**Figure 1:**
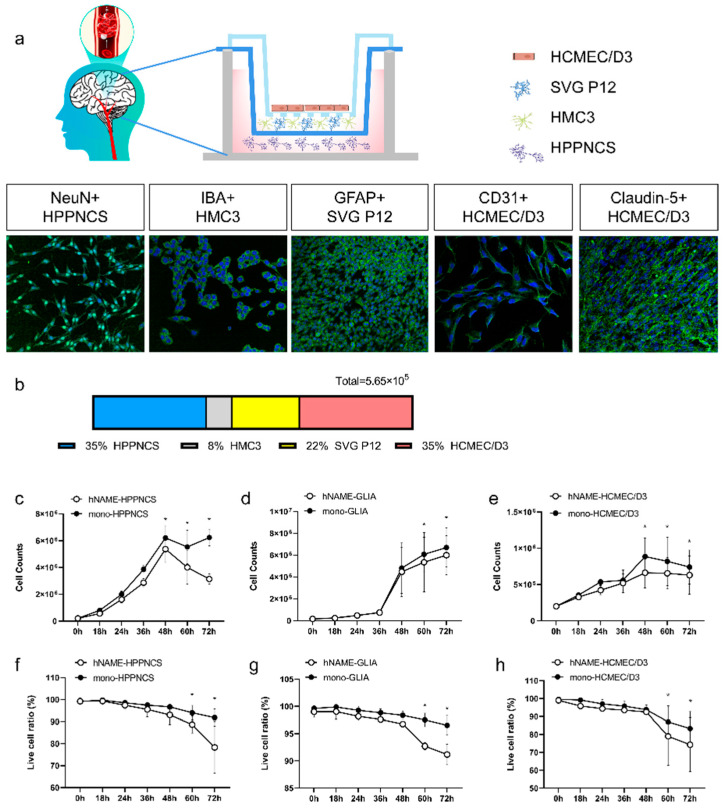
Composition of the micro-organ model (hNAME). (**a**) Human-derived neurons, astrocytes, microglia, and cerebral microvascular endothelial cells constitute a micro-organ of the human brain microenvironment in an in vitro model. (**b**) Four cell composition ratios. (**c**–**e**) Cell counts in the micro-organ microenvironment model, compared with monolayer culture under standard conditions. (**f**–**h**) Live cell ratio of the micro-organ microenvironment model, compared with that in a monolayer culture under standard conditions. * *p* < 0.05.

The lower chamber was plated with 2 × 10^5^ human hippocampal neurons (N) expressing the neuron-specific marker NeuN. The medium chamber was plated with 1.2 × 10^5^ human astrocytes (A) of SVG P12 and 4.5 × 10^4^ human microglia (M) of HMC3, expressing glial fibrillary acidic protein (GFAP) and the ionic calcium-binding adapter molecule (IBA1), respectively. The upper chamber was plated with 2 × 10^5^ HCMEC/D3 human microvascular endothelial cells (E) expressing the vascular-specific protein marker CD31, as shown in Figure 1a. As shown in Figure 1b, 35% of the total volume was composed of neurons, followed by 22% astrocytes, 8% microglia, and 35% endothelial cells. These four cell types were identified as hNAME, using their initials, and constituted a significant portion of human cells.

We compared the number of cells and their ability to survive in an hNAME culture with monolayer culture systems at various time intervals (18, 24, 36, 48, 60, and 72 h) in a standard culture environment (incubator with 95% oxygen and 5% carbon dioxide at 37 °C) after connecting the plate. Both cultures showed a decline in cell count and viability after 48 h, indicating that the cells were not healthy after the standard 48 h culture period. We recommend not extending the modeling period beyond 48 h. Notably, the neurons and vascular endothelial cells in the hNAME group had lower numbers and viability than those in the monolayer group, as shown in Figure 1c–h.

### 2.2. Comparison of hNAME and Monolayer Culture at Different OGD Time

**Figure 2:**
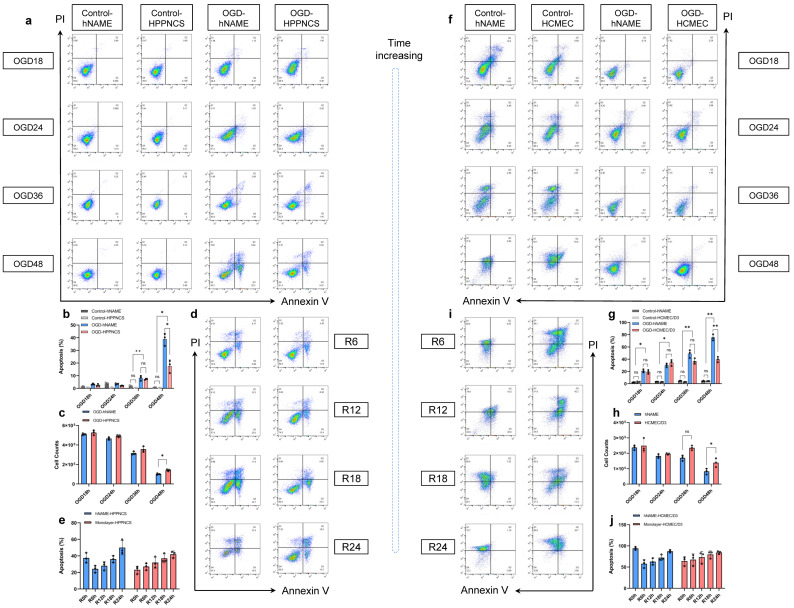
Differences in cell injury after oxygen-glucose deprivation (OGD) between the hNAME model and the monolayer culture. (**a**) Neuron apoptosis rate in hNAME and monolayer cells under normal and OGD environments for different durations. (**b**) Statistical results of neurons in group differences. (**c**) Cell counts of neurons in group differences. (**d**) Differences in neuronal apoptosis rates between hNAME and monolayer culture at different reoxygenation times after OGD for 48 h. (**e**) Statistical results of apoptosis rates in two groups at different reoxygenation times. (**f**) The apoptosis rate of microvascular endothelial cells in hNAME and monolayer cells under normal and OGD environments for different durations. (**g**) Statistical results of microvascular endothelial cells in terms of group differences. (**h**). Cell counts of microvascular endothelial cells in terms of group differences. (**i**) Differences in the microvascular endothelial cells apoptosis rates between hNAME and monolayer culture at different reoxygenation times after OGD for 48 h. (**j**) Statistical results of apoptosis rates of microvascular endothelial cells in two groups at different reoxygenation times. * *p* < 0.05, ** *p* < 0.01.

After the initial experiment, the viability of neurons and the number of living cells decreased after 60 and 72 h of standard culture, respectively. Cells within the 18–48 h range were chosen to construct the OGD model. The results showed that the neuronal apoptosis rates in the OGD 18, 24, 36, and 48 h hNAME models were 3.25 ± 0.54%, 3.51 ± 0.57%, 8.17 ± 1.98%, and 38.90 ± 4.79%, respectively. The apoptosis rates of the monolayer neurons were 2.52 ± 0.86%, 2.04 ± 0.21%, 7.24 ± 0.54%, and 17.56 ± 4.99%, respectively. The rate of neuronal apoptosis increased significantly as the duration of OGD increased (*p* < 0.01). The apoptosis rates of hNAME and monolayer neurons at 36 and 48 h of OGD were higher than those of the control group (standard culture). This difference was statistically significant (*p* < 0.05), with the apoptosis rate being highest at 48 h of OGD (*p* < 0.01), as shown in Figure 2a. The hNAME model exhibited more severe apoptosis than the monolayer culture system (mean difference = 21.34, 95% Cl = 5.06–37.63; *p* = 0.020) and cell viability (mean difference = −4.7 × 10^4^, 95% Cl = −84997 to −9003; *p* = 0.028). There were no significant differences at the other OGD durations (*p* > 0.05), as shown in Figure 2b,c.

We compared the apoptosis rates of monolayer neurons and hNAME cells at different reoxygenation time points after 48 h. An ANOVA revealed interactions between the different reoxygenation times in both groups (*p* = 0.004). After 48 h of OGD, the neuronal apoptosis rates at reoxygenation for 0 h (R0), 6 h (R6), 12 h (R12), 18 h (R18), and 24 h (R24) were 23.1 ± 4.59%, 27.06 ± 4.25%, 31.9 ± 6.71%, 36.9 ± 5.46%, and 41.9 ± 4.09%, respectively, as shown in Figure 2d. Unlike the trend of monolayer neurons, where gradually worsening damage was observed with increasing reoxygenation time, the apoptosis rate of hNAME neurons decreased at R6 (24.2 ± 4.21%) compared with R0 (37.5 ± 6.01%). Subsequently, the severity of the injury increased with the increase in reoxygenation time (R12 = 27.9 ± 5.0%, R18 = 36.0 ± 4.0%, R24 = 50.0 ± 8.70%, *p* = 0.002). The overall trend presented a biological characteristic similar to ischemic-reperfusion brain injury, which is first alleviated and then worsened with oxygenation, as shown in Figure 2e.

Next, we observed the differences between hNAME- and monolayer-cultured human microvascular endothelial cells. The apoptosis rates of HCMEC/D3 in hNAME with OGD at 18, 24, 36, and 48 h were 20.83 ± 2.54%, 50.72 ± 0.83%, 75.4 ± 2.83%, and 95.1 ± 2.56%, respectively. The HCMEC/D3 monolayer exhibited apoptosis rates of 19.28 ± 3.12%, 33.4 ± 2.21%, 57.6 ± 2.65%, and 89.3 ± 3.98%. All apoptosis rates in the OGD groups were significantly higher than those in the control groups (*p* < 0.05). The rates increased with longer OGD durations (*p* < 0.01), a trend consistent with that observed in neurons, as shown in Figure 2f. However, unlike neurons, HCMEC/D3 cells were more prone to damage than HPPNCS. After 18 h of OGD, the apoptotic rate of HCMEC/D3 cells was over 20%, whereas the neurons remained mostly unaffected. During a stroke, vessels are the first to undergo necrosis and find it difficult to recover, this being followed by neuronal apoptosis. This is consistent with our results that HCMEC/D3 apoptosis occurs first and is worse than in hNAME neurons, similar to the previous report [18]. We found that the HCMEC/D3 apoptosis rate in the hNAME group was higher than that in the monolayer group after comparing both OGD groups. However, the difference between the hNAME groups was significantly higher than that between the monolayer groups after 48 h (mean difference = 35.64, 95% Cl = 19.8–51.48; *p* = 0.0028), as shown in Figure 2g. The viability of HCMEC/D3 cells was also significantly different between the hNAME and monolayer groups at 48 h (mean difference = −5.6 × 10^4^, 95% Cl = −1.05 × 10^5^ to −0.79 × 10^4^; *p* = 0.037), as shown in Figure 2h.

We also observed changes in the HCMEC/D3 cells at different reoxygenation times. The difference between the monolayer culture and hNAME also existed in the upper chamber of endothelial cells, as shown in Figure 2i,j. The monolayer HCMEC/D3 sustained increasing damage over time following OGD for 48 h and reoxygenation. However, HCMEC/D3 cells in the hNAME group did not experience the same worsening. There was no significant increase in the rate of apoptosis at the R6h time point after OGD for 48 h compared to R0h in the hNAME group. HCMEC/D3 exhibited relief at OGD48/R6h, similar to that seen in the HPPNCS of hNAME.

### 2.3. The Difference in Inflammatory Factors between hNAME and Monolayer Culture

**Figure 3:**
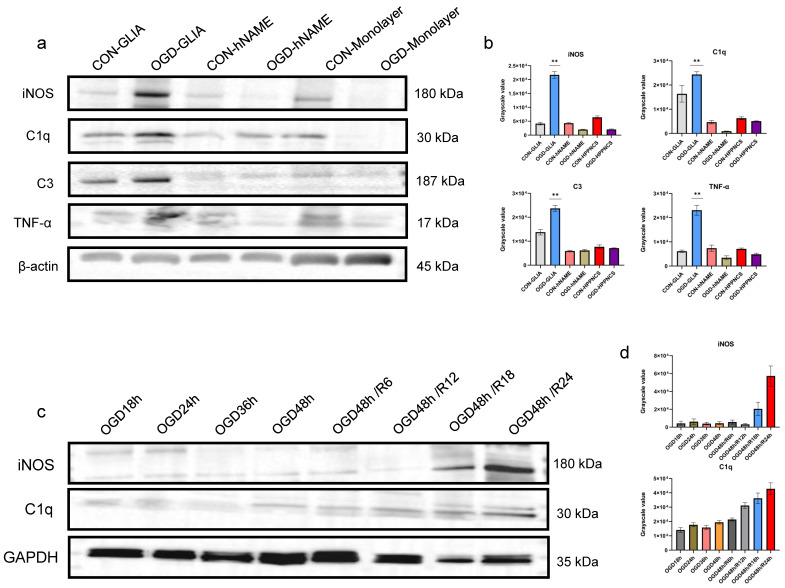
Quantitative results of inflammatory factors. (**a**,**b**) C1q, C3, TNF-α, and iNOS are highly expressed in the glial cell layer of hNAME rather than in monolayer cells. (**c**) Representatives of the inflammatory factors, iNOS and C1q, had the highest expression at OGD 48 h and reoxygenation at 24 h. (**d**) Statistical results of c. ** *p* < 0.01.

In addition to checking for cell apoptosis, we examined the levels of inflammatory markers such as C1q, C3, TNF-α, and the inducible nitric oxide synthase (iNOS). These markers are associated with pro-inflammatory microglia and astrocytes. These factors were mainly present in the microglia and astrocyte chambers of the hNAME group; however, there were no notable differences in the neurons located in the hNAME monolayer, as shown in Figure 3a,b. In addition, the levels of inflammatory factors increased with the duration of OGD and reoxygenation. This indicated that the inflammatory response worsened with prolonged hypoxia and deteriorated more with longer reoxygenation times, as shown in Figure 3c,d.

### 2.4. Characterizations of hUMSCs

**Figure 4:**
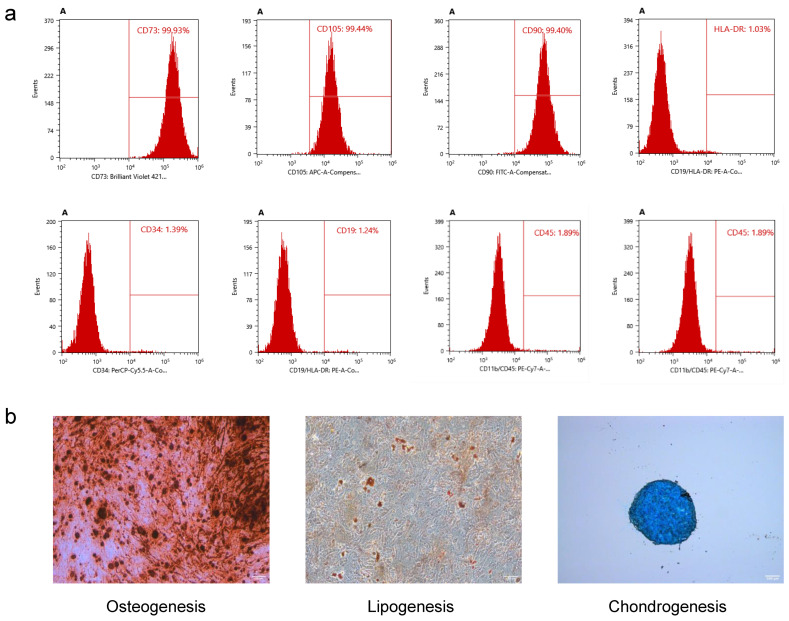
Flow cytometry and the differentiation ability of hUMSCs. (**a**) CD73, CD105, and CD90 stainings were positive, HLA-DR, CD34, CD45, CD11b, and CD90 stainings were negative. (**b**) The stemness ability of hUMSCs and the cell morphology and differentiation of HUMSCs into fat, bone, and cartilage. The scale bar is 100 μm.

The hUMSCs grew into the shape of a long spindle, and the vortex was in good growth condition. The flow cytometry characterization of the hUMSCs showed that the cells were positive for CD73, CD105, and CD90 at 99.9%, 99.7%, and 99.8%, respectively. The HLA-DR, CD34, CD45, CD19, and CD11b antibodies showed negative expressions at 1.03%, 1.39%, 1.89%, 1.24%, and 1.90%, respectively, as shown in Figure 4a.

The hUMSCs showed positive staining characteristics of adipogenic, osteogenic, and chondrogenic differentiation (lipid droplets, calcium nodules, and cartilage polysaccharides), as shown in Figure 4b.

### 2.5. Effect of hUMSCs on hNAME and Monolayer Culture

These results demonstrated that the hNAME model can simulate the pathophysiological characteristics of HIBI through OGD, including cell apoptosis and inflammatory factor release. Furthermore, we evaluated the value of hNAME in stem cell therapy. 

**Figure 5:**
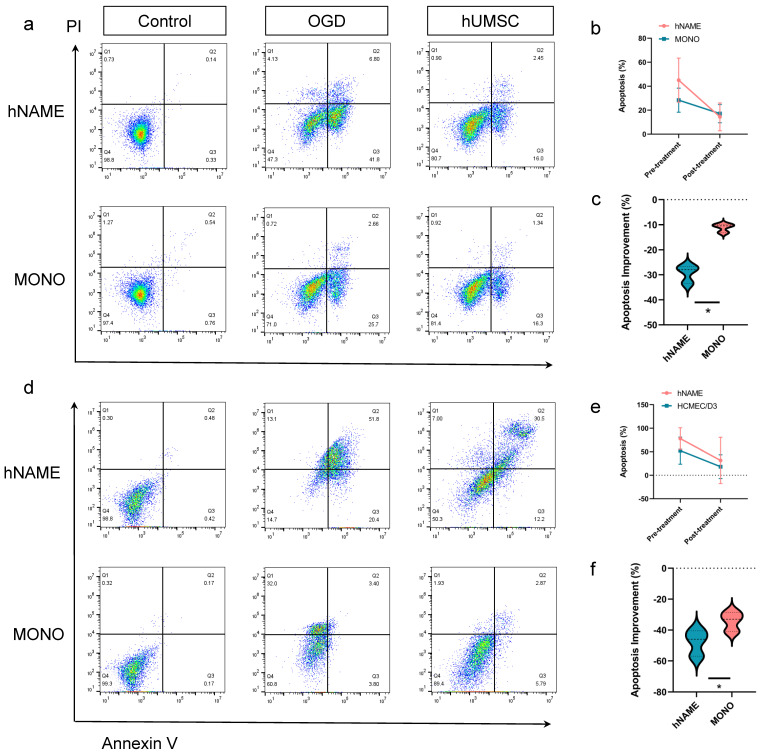
(**a**) Comparison of apoptosis rates between hNAME neurons and monolayer neurons after hUMSC treatment. (**b**) Line graph of the apoptosis rate change between hNAME neurons and monolayer cultured neurons. (**c**) Comparison of the nerve injury improvement between the hNAME and monolayer neurons after hUMSC treatment. (**d**) Comparison of apoptosis rates between hNAME and monolayer brain microvascular endothelial cells after hUMSC treatment. (**e**) Line graph of apoptosis rate changes between hNAME and monolayer cultured brain microvascular endothelial cells. (**f**) Comparison of the nerve injury improvement between the hNAME and monolayer brain microvascular endothelial cells after hUMSC treatment. * *p* < 0.05.

The apoptosis rate at post-treatment (14.4 ± 4.6%) in the hNAME model was greatly reduced compared to that at pretreatment (45.0 ± 7.4%). The apoptosis rate of monolayer neurons was also significantly reduced (28.3 ± 4.0% in the OGD group, 17.1 ± 3.1% in the hUMSC group), as shown in Figure 5a. The GLM results showed that the post-treatment apoptosis rate differed significantly between the hNAME and monolayer groups when the apoptosis rate was corrected (adjusted mean difference = −13.45, *p* = 0.04), as shown in Figure 5b. However, the improvement of neuronal apoptosis in hNAME (29.3 ± 3.5%) was higher than that in the monolayer neuron (11.2 ± 1.7%) post-treatment, as shown in Figure 5c. 

The apoptosis rate of HCMEC/D3 cells in hNAME was higher than that in the monolayer at pretreatment; however, there was no difference between the groups after treatment. The GLM results showed that the post-treatment apoptosis rate was significantly different after collecting the model (adjusted mean difference = −19.38, 95% Cl = −38.2–−0.5, *p* = 0.04), as shown in Figure 5d,e. The improvement of apoptosis in hNAME was also superior to that in the monolayer group (mean difference = 15.23, 95% Cl = 0.66–29.80, *p* = 0.04), as shown in Figure 5f.

## 3. Discussion

Our study utilized immortalized human hippocampal neurons, astrocytes, microglia, and vascular endothelial cell lines to create in vitro models that mimic central nervous system diseases, using a three-layer stereo structural culture system. This model is advantageous owing to its ease of use, quick setup time, and consistent results. Additionally, this study addresses the issue of species differences that arise in experimental studies. This model is stable and is appropriate for studying various neurological disorders.

Although primary cells can represent more in vivo biological characteristics than immortalized cell lines, they require more time to grow and culture. On the one hand, even under optimal growth conditions, their growth potential is limited. On the other hand, primary cells from different donors have different inflammatory responses and metabolic abilities, and their biological characteristics may change with wear, resulting in poor experimental repeatability. Most importantly, the separation of primary cells in the human body is ethically limited, and the possibility of access is minimal. The multiple-culture model has more advantages than other models that are used to study neurological disorders in vitro. The most obvious benefit is the presence of neurons, astrocytes, microglia, and vascular endothelial cells in the same cell culture medium, allowing researchers to investigate the cell-to-cell interactions. Although many in vitro models exist, such as organoids [21,22], 3D scaffolds [23], and microfluidics [24], our co-culture system is simple to construct, with an easy cell culture workflow. The hNAME device is convenient for separation and arbitrary combination, and the cells are layered for bulk RNA sequencing, similar to the process used in single-cell RNA sequencing. In addition, the hNAME system provides enough cells to complete Western blotting, flow cytometry, and other experiments at the same time. More importantly, this in vitro model has a stable cell state, ensuring good repeatability of the experimental results.

Monolayer-cultured cells are generally inexpensive and are easy to create; however, it is impossible to use them to replicate the complexity of the in vivo system, which limits their biological relevance. Organoids and neurospheres are examples of in vitro models that rely on the growth of stem cells or differentiated cells, which naturally form a complex, multicellular environment [25]. Nonetheless, limitations may arise due to the high variability and ease with which necrotic cores can form [26]. An important factor to consider is that brain-like organs grown from stem cells originate from neuroectodermal cells, which lack microglia of mesodermal origin [27,28,29]. This can pose a challenge when studying the interactions between neurons, astroglia, and microglia in the environment. Our hNAME model offers a significant advantage as it includes microglia and simulates the immune microenvironment of the brain, based on the proportion of human brain cells. Notably, there were no significant differences in the number and quality of hNAME neurons and monolayer neurons under standard culture conditions. However, the damage inflicted on hNAME neurons and vascular endothelial cells during modeling was considerably more severe than that experienced by monolayer neurons and vascular endothelial cells. Research has shown that astrocytes and microglia in low-oxygen environments release substances that cause inflammation, which can exacerbate the damage to neurons and blood vessels. Interestingly, the severity of injury decreased in the early stage of reoxygenation (6 h) after 48 h, which may be explained by microglial autophagy. Autophagy, when appropriate, can have a protective effect on ischemic nerve tissue. However, excessive autophagy can also lead to cell death [30,31].

The repair levels of hNAME neurons and microvascular endothelial cells were higher than those in the monolayer after hUMSC treatment, which may be due to the participation of microglia in neurogenesis. Studies have shown that microglia in the dentate gyrus alter their phenotype and transcription levels to release factors related to neurogenesis and vascular endothelial growth factors [32]. Furthermore, the suppression of microglial gene expression reduced the survival of neuroblasts in the adult hippocampus [32]. Previous studies suggest that MSC exosomes and their vesicles may have the ability to alleviate cerebral ischemic-reperfusion injury, regulate neuroinflammation, prevent cell death, and modify microglial behavior and astrocyte alterations [33,34,35]. In this study, thehUMSCs caused damage to neurons, and cerebral microvascular endothelial cells in the hNAME model greatly improved compared to the monolayer cultures. The types of microglia and astrocytes modulated by hUMSCs may be the primary mechanism underlying the beneficial recovery effect. Our model demonstrated the importance of cell interactions between microglia and astrocytes in an in vitro brain model.

This model may be applicable to other models of central nervous system diseases. This study selected only the most common cerebrovascular diseases and stem cells for intervention to demonstrate that hNAME is an ideal in vitro model that simulates the central immune microenvironment.

## 4. Materials and Methods

### 4.1. hNAME Model Preparation

A three-layer, three-dimensional co-culture system was created by connecting a six-well Transwell cell culture plate to a 12-well Transwell membrane. First, 2 × 10^5^ human cerebral microvascular endothelial cells (HCMEC/D3, Jennio Biotech Co., Ltd., Guangzhou, China) were seeded in the upper 12-well Transwell membrane (upper chamber) and cultured in a routine incubator at 37 °C for two days until tight junctions between the cells were formed, to obtain a blood-brain barrier. On the second day, 1.2 × 10^5^ astrocytes (SVG P12, BLUEFBIO^TM^, Shanghai, China) and 4.5 × 10^4^ human microglia (HMC3, BLUEFBIO^TM^, Shanghai, China) were seeded in the medium layer of a six-well Transwell membrane (medium chamber), and 2 × 10^5^ human hippocampal neurons (HPPNCS, BLUEFBIO^TM^, Shanghai, China) were seeded in the bottom layer (lower chamber). All four cell types were inoculated into high-glucose Dulbecco’s Modified Eagle Medium (DMEM), containing 10% fetal bovine serum (Procell Life Science&Technology Co., Ltd., China) with 1% penicillin and streptomycin (Procell Life Science&Technology Co., Ltd., China, China), for 20–24 h to achieve normal cell morphology. The three-dimensional multicell co-culture model was subjected to OGD on the third day.

### 4.2. Monolayer Cell Culture

Consistent with the plating time of the hNAME model, the single-layer culture system only seeded 2 × 10^5^ HPPNCS in a six-well plate as a monolayer neuron culture, with another six-well dish seeding 2 × 10^5^ HCMEC/D3 as a monolayer vascular endothelial cell culture. The media used was high-glucose DMEM, same as that in the hNAME model.

### 4.3. Oxygen-Glucose Deprivation

hNAME model cells and monolayer cultured cells were removed from the conventional incubator, then the medium was replaced with Earle’s Balanced Salt Solution (EBSS) sugar-free medium, and the cell model was placed at 37 °C, 95% N2, and 5% CO_2_ hypoxic incubators for 18, 24, 36, 48, and 60 h. After OGD, the two models were removed, and the medium was replaced with DMEM high-sugar medium for reoxygenation in a conventional incubator for 6, 12, 18, or 24 h. Cells were collected at different time points for subsequent testing.

### 4.4. Preparation of hUMSCs 

hUMSCs were extracted using the tissue block culture attachment method, as previously reported [19,20]. The preparation of P5 hUMSCs was conducted in a GMP facility following good clinical practice (GCP) guidelines. The identification of hUMSCs was detected by flow cytometry (BD Biosciences, FACSAria2, USA). The antibodies used in this experiment included CD73-BV421 (cat.562430, BD Biosciences, USA), CD105-APC (cat. 562408, BD Biosciences, USA), CD90-FITC (cat.555595, BD Biosciences, USA), HLA-DR-PE (cat. 555812, BD Biosciences, USA), CD34-PerCP-Cy™5.5 (cat. 347203; BD Biosciences, USA), and CD45-FITC (cat.555482; BD Biosciences, USA). Osteogenic differentiation medium (catalog#05465, STEMCELL Technologies, China), adipogenic differentiation medium (catalog#05412, STEMCELL Technologies, China), and chondrogenic differentiation medium (catalog#05455, STEMCELL Technologies, China) were used to assess the osteogenic, adipogenic, and chondrogenic differentiation potential of hUMSCs, following the manufacturer’s instructions. Oil Red O solution (O1391, Sigma-Aldrich, USA), Alizarin Red S (A5533, Sigma-Aldrich, USA), and Alcian Blue staining solution (TMS-010-C, Sigma-Aldrich, USA) were used for adipogenic, osteogenic, and chondrogenic staining, respectively, according to the manufacturer’s instructions.

### 4.5. Flow Cytometry

The cells were detached from the plate by an ethylenediaminetetraacetic acid-free trypsin solution, and the supernatant was discarded after centrifugation. The cells of different groups were collected into the flow tube and resuspended with 100 μL Annexin V binding buffer. Next, 5 μL Annexin V reagent and 10 μL Propidium Iodide solution were added, and the cells were incubated at room temperature for 15 min away from light. After the dyeing step, 200 μL Annexin V binding buffer was added and diluted before testing. The early and late apoptosis rates were determined by measuring the number of positive cells in the APC and PI channels.

### 4.6. Western Blot

Protein extraction: (1) The cells were centrifuged, the supernatant was discarded, RIPA lysis buffer was added, and the mixture was cracked on ice for 30 min and centrifuged at 4 °C 13,000rpm for 15 min. (2) The supernatant (protein solution) was put into the EP tube, and 4 μL was taken for BCA quantitative analysis. (3) A loading buffer of 1/3 of the supernatant volume was added into the EP tube, placed in a metal bath at 85 °C, and incubated for 10 min. Finally, BCA quantitative analysis of the sample protein concentration was performed.

Western blotting: (1) The running and trans buffers were configured. (2) The sample amount was calculated according to the protein concentration and was not less than 10 μg per well. (3) The following settings were applied: 80 V, 300 mA, 15 min; 150 V, 300 mA, 60 min constant pressure running. (4) The PVDF membrane was cut according to the size of the internal parameters and target proteins. (5) The glue was cut according to the size of the internal parameters and target proteins. (6) The transfer box was installed according to the principle of “glue against black, PVDF membrane against white”. (7) The settings were set at 600 V and 300 mA, the transfer time was determined according to the protein molecular weight size, and the sample was cooled down in the ice box. (8) The sample was sealed with 40 ml milk for 2 h. (9) Briefly, 1 × TBST film washing was performed for 5 min. (10) The sample was diluted with an antibody diluent in accordance with the dilution ratio of the antibody instructions at 4 °C overnight. (11) Then, 1 × TBST film washing was performed three times for 10 min each time. (12) The secondary antibody was diluted in accordance with the antibody instructions and incubated for 2 h at room temperature and away from light. (13) The film was washed with TBST three times for 10 min each time. (14) Finally, the luminescent liquid was configured, and photos were taken using an imaging device.

### 4.7. Immunostaining

After collection, the cell culture medium was discarded, and the cells were fixed with paraformaldehyde (PFA). Then, the cells were washed three times with PBS. Next, the cells were treated with an immunostaining permeable solution (Triton X-100) for 10–15 min and washed thrice with PBS. Finally, the cells were sealed for 10–15 min. 

The corresponding proportion of antibodies was mixed with the primary antibody diluent to prepare the primary antibody dye and then kept in the refrigerator at 4 °C overnight. The primary antibody was retrieved the following day, and the cells were washed three times with PBS. The secondary antibody diluent was used to proportionally dilute the corresponding secondary antibodies, which were incubated at room temperature in the dark for 1.5 h, followed by five washes with PBS. Finally, DAPI (Abcam 104139) was added and incubated at room temperature for 5 min. A CQ1 dual-rotary high-resolution imaging system was used to complete this process. The following antibodies were used: GFAP (CST 3670), anti-NeuN (CST 24307), IBA-1 (Abcam EPR16588), and CD31 (Abcam EPR3094).

### 4.8. Cell Count and Viability

A cell suspension was prepared by removing the hNAME and monolayer cultured cells from the well plate, discarding the medium, washing the cells once with PBS, and adding 500 μL of trypsin for digestion. The well plate was placed into a cell incubator at 37 °C to aid digestion and removed after 2–3 min. Two times the medium volume (1 mL) was added to neutralize the digestion for both hNAME and monolayer cultured cells. The cell suspension was transferred into separate 15 mL centrifuge tubes and centrifuged at 1000 rpm for 5 min. After centrifugation, the supernatant was discarded, and the cell sediment was retained. Then, 1 mL of DMEM medium containing 10% fetal bovine serum was added to resuspend the cells evenly. A sample of cell stock (10 μL) was taken from both hNAME and monolayer cultured cell systems and mixed with Trypan Blue stain (Invitrogen T10282, Thermo Fisher Scientific, USA). The suspension was mixed by pipetting slowly up and down ten times before slowly dropping the resulting cell suspension from the edge of a counting slide (Invitrogen C10312, Thermo Fisher Scientific, USA), ensuring that the gap between the mounting plate and the cover plate was filled. An automated cell counter (Invitrogen Countess 3 AMQAX2000, Thermo Fisher Scientific, USA) was used to count the live cells and viability. These measurements were integrated over three repetitions to calculate an average value. 

### 4.9. Statistical Analysis

Each experiment was conducted with at least three biological replicates to ensure accuracy, and each biological replicate had a minimum of three technical replicates. We utilized SPSS 23.0 (SPSS Inc., Chicago, IL, USA) and set the significance threshold at α = 0.05. A generalized linear model (GLM) was employed to analyze the continuous and categorical variables. We tested for differences between the groups using the main effects if the outcomes did not show a significant interaction.

## 5. Conclusions

In this study, we provide an in vitro cell model that is easy to construct, low in cost, is less time-consuming, stable, and can highly simulate the human brain immune microenvironment. Taking common hypoxic-ischemic brain injury as an example, we demonstrate the value of the micro-organ of the human brain model because of microglia and astrocyte participation, showing that the OGD injury was more severe compared with general culture, while the stem cells treatment was better.

## Data Availability

Please contact the corresponding author to get data.
